# Flexible Retrospective Phase Stepping in X-Ray Scatter Correction and Phase Contrast Imaging Using Structured Illumination

**DOI:** 10.1371/journal.pone.0078276

**Published:** 2013-10-31

**Authors:** Han Wen, Houxun Miao, Eric E. Bennett, Nick M. Adamo, Lei Chen

**Affiliations:** 1 Imaging Physics Laboratory, Biochemistry and Biophysics Center, National Heart, Lung and Blood Institute, National Institutes of Health, Bethesda, Maryland, United States of America; 2 Center for Nanoscale Science and Technology, National Institute of Standards and Technology, Gaithersburg, Maryland, United States of America; Virginia Tech, United States of America

## Abstract

The development of phase contrast methods for diagnostic x-ray imaging is inspired by the potential of seeing the internal structures of the human body without the need to deposit any harmful radiation. An efficient class of x-ray phase contrast imaging and scatter correction methods share the idea of using structured illumination in the form of a periodic fringe pattern created with gratings or grids. They measure the scatter and distortion of the x-ray wavefront through the attenuation and deformation of the fringe pattern via a phase stepping process. Phase stepping describes image acquisition at regular phase intervals by shifting a grating in uniform steps. However, in practical conditions the actual phase intervals can vary from step to step and also spatially. Particularly with the advent of electromagnetic phase stepping without physical movement of a grating, the phase intervals are dependent upon the focal plane of interest. We describe a demodulation algorithm for phase stepping at arbitrary and position-dependent (APD) phase intervals without assuming *a priori* knowledge of the phase steps. The algorithm retrospectively determines the spatial distribution of the phase intervals by a Fourier transform method. With this ability, grating-based x-ray imaging becomes more adaptable and robust for broader applications.

## Introduction

X-ray phase contrast imaging and scatter correction are both being developed for the benefit of medical diagnosis, where x-ray modalities account for 70% of the diagnostic imaging procedures in the US [1]. An interesting converging point of the two fields is a class of methods that use gratings or grids to introduce a periodic modulation into the x-ray wave, either by simple geometric shadowing or coherent wave interference effects [2]–[6]. Phase contrast relates to the distortion of the periodic fringes by refractive bending of the x-rays in the imaged object, while scattering in the object causes a loss of the fringe amplitudes in excess of the conventional intensity attenuation [5], [7]. Several methods have been proposed to retrieve the amplitude and the positions (phase) of the fringes in the two areas of application. The quickest method requires just a single image, where the phase value is measured by the displacement of the fringes, and the amplitude is measured by the intensity oscillation in a fringe period. Such measurements can be made efficiently over the entire image through Fourier analysis [5], [8], [9], or directly in the real space [10]. However, a limitation of single image analysis is that the spatial resolution of the measurements is no finer than the fringe period, which is at least 3 times the resolution of the imaging device in order for the fringes to be clearly resolved.

**Figure 1:**
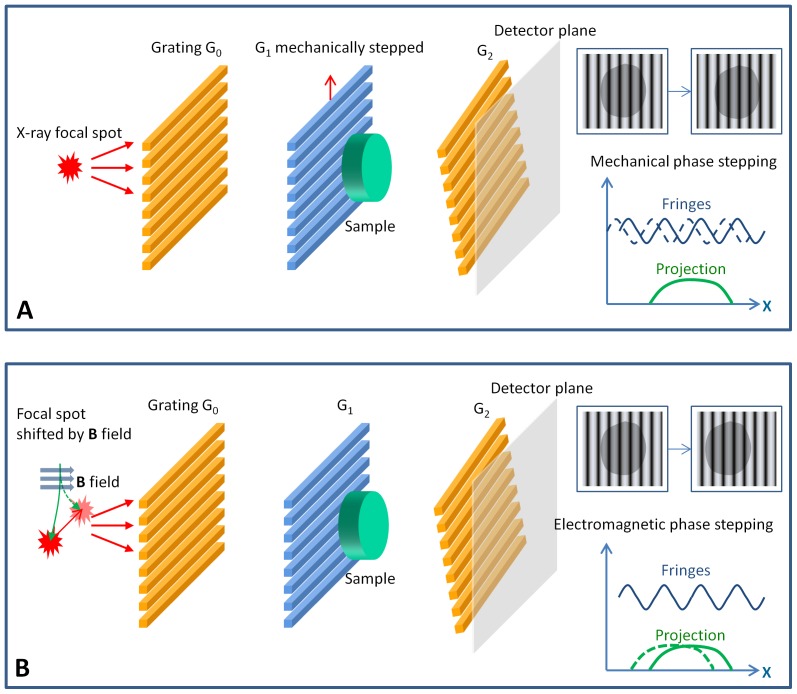
Phase stepping procedures measure the distortion and scattering of a grating-modulated wavefront. These are examples of grating-based phase contrast imaging devices, where the combination of an absorption grating G_0_ and a phase grating G_1_ produces primary interference fringes which are masked by a slightly rotated absorption grating G_2_, resulting in broader moiré fringes that can be resolved by the detector. (**A**) In mechanical phase stepping, the phase grating G_1_ is moved in-plane perpendicular to the grating lines, at increments of a fraction of the grating period. This creates incremental shifts of the moiré fringes, and equivalently a periodic oscillation of the intensity at each detector pixel. The amplitude and phase of this oscillation encode the information about the distortion and scattering of the wavefront as it propagates through the sample. These are retrieved by an adaptive algorithm which is the focus of this paper. (**B**) In motionless electromagnetic phase stepping, the focal spot of the x-ray source is shifted with an externally applied magnetic (***B***) field, which results in a relative movement between the projection image of the sample and the moiré fringes. The images are digitally shifted to re-align the projections while the moiré fringes appear to move, effectively synthesizing the phase stepping process. In this example, the applied magnetic field deflects the electron beam in the x-ray tube, shifting its impact point on the anode target where x-rays are emitted.



This problem is solved by the phase stepping method at the cost of acquiring multiple images [11]. In phase stepping, a grating is moved perpendicular to its lines in uniform increments while images are taken at each step. This ideally results in uniform shifts of the fringes (Fig. 1A). Equivalently, it produces a periodic oscillation of the intensity at each pixel in the image. In the temporal domain, this procedure provides several points along the intensity oscillation curve at uniform phase intervals. If the phase interval is an integer fraction of a complete cycle, i.e. 2π/*N* where *N* is the total number of steps, then the intensity at a location **r** in the *n*th image can be expressed as
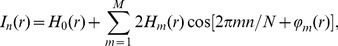
(1)where *H_m_* and *φ_m_* are the amplitude and phase of the *m*th order harmonic of the intensity oscillation. By considering multiple harmonics, this expression covers any possible periodic waveform of the oscillation. The harmonic amplitudes are the Fourier coefficients of the series of intensities *I_n_*, and thus can be calculated by an inverse Fourier transform:

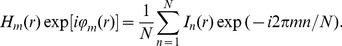
(2)This algorithm was developed in wave-front-measuring interferometry [11] and subsequently applied to x-ray phase contrast [2], [4], [12], [13] and scatter correction [6].

However, in practical settings there are often drifts and errors in the position and orientation of the grating. Then, the phase intervals become uncertain and may vary spatially with position. Furthermore, electromagnetic phase stepping (EPS) has recently been developed to eliminate all mechanical motion [14], where phase stepping is synthesized by a relative movement between the projection of the object and the fringe pattern (Fig. 1B). The relative movement is realized by electromagnetically shifting the focal spot of the cone beam, and it is thus dependent on the position of the object, or more specifically the focal plane of image reconstruction. Consequently, the phase intervals become variable and not limited to integer fractions of 2π. In all these cases, the intensity of the *n*th image needs to be expressed in a more generalized way as
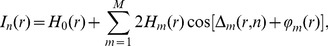
(3)where Δ*_m_*(**r**, *n*) is the phase shift applied by the phase stepping process and can be arbitrary and position dependent (APD). The problem we address is how to retrieve the harmonic oscillation amplitude *H_m_* and phase *φ_m_* from such arbitrary phase shifts.

The solution for the relatively ideal conditions in wave-front-measuring interferometry has been described, under the assumptions that the phase increments in the phase stepping process is globally uniform, and the fringes are well defined in the entire image [15]. However, in diagnostic imaging situations the conditions are usually less ideal and can violate both assumptions. Specifically, the phase shifts can be position dependent, and the fringe visibility in areas of high attenuation or scattering is degraded. Here we extend the special solution for wave-front characterization to a more general and adaptable one for x-ray imaging, without making the above assumptions. We demonstrate its use in x-ray phase contrast imaging of biological samples using electromagnetic phase stepping.

## Methods

### Processing Algorithm for Arbitrary, Position-Dependent Phase Steps

The algorithm consists of two steps, including determining the applied phase shifts Δ*_m_*(**r**, *n*) for the images in the phase stepping set, and calculating the oscillation amplitude *H_m_*(**r**) and phase *φ_m_*(**r**). The second step will be described first using the applied phase shifts as *a priori* information. From Eq. (3), the images can be expanded into a linear combination of complex amplitudes *A_m_*:
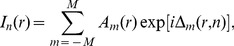
(4)where *A_m_* relates to the harmonic amplitude *H_m_*(**r**) and phase *φ_m_*(**r**) by
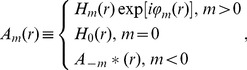
(5)with * indicating the complex conjugate, and the positive and negative harmonic orders are conjugate of each other such that



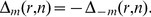
(6)The goal is to solve for *A_m_*. For this purpose the total number of images in the phase stepping set *N* should be equal to or greater than the number of unknowns, which is 2*M*+1. Generally *N* >2*M*+1, in which case the unknowns *A_m_* are determined by a least-squares method that minimizes the error function for every location (***r***) as
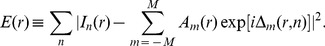
(7)


The solution can be expressed in matrix form as
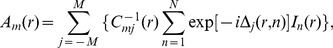
(8)where the matrix *C* is calculated from the applied phase shifts Δ*_m_*(**r**, *n*) by



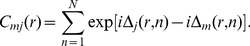
(9)For efficient computation, the solution for *A_m_* is written as linear combinations of the acquired images in the phase stepping set,
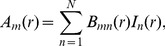
(10)where the coefficients of the linear combinations are



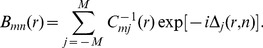
(11)It will be shown below that the calculation of the coefficients *B_mn_*(***r***) is done at a reduced resolution to improve computation speed, and then interpolated back to the full detector resolution and used as inputs in Eq.(10) to obtain the complex amplitudes *A_m_* at full resolution. Once the *A_m_*‘s are obtained, the amplitudes and phases of the various harmonics of the intensity oscillation at each pixel is expressed as the inverse of Eq. (5):
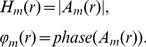
(12)


Now we describe how to determine the actual phase increments for all images in the phase stepping set, i.e. the applied phase shifts Δ*_m_*(**r**, *n*), without *a priori* knowledge. The basic idea is to treat the applied phase shifts as free functions of position, and measure them from the acquired images using a Fourier-transform method [5], [15]. The Fourier method was first developed for interferogram analysis [8], [16]. The application of the method requires the presence of a spatial carrier frequency in the real space domain, i.e. a fringe pattern in the image. In grid-based scatter imaging and scatter correction, the projection of the absorption grids is a periodic fringe pattern which provides the carrier frequency modulation. In phase-contrast imaging using high line density gratings, the grating periods are often smaller than the resolution of the detector, requiring broader moiré fringes to be formed in order to detect the phase shifts. This is accomplished by a slight rotation of one of the gratings away from the perfect alignment. An example is illustrated in the systems in Fig. 1, in which the absorption grating G_2_ is rotated slightly around the beam axis relative to the G_0_ and G_1_ gratings, leading to moiré fringes on the detector screen. The frequency of the fringes is obtained in data processing from calibration images without any sample. The 2D Fourier transform of the calibration image contains discrete peaks located at integer multiples of the carrier frequency [8]. The position of the first-order peak is identified in the Fourier domain, and provides the carrier frequency.

The spatial carrier frequency must be high enough to adequately separate the components of various harmonic orders in the Fourier domain [16]. In real space it means that the fringes are dense enough such that the periods do not vary drastically within the distance of a single period. In grating-based imaging, the applied phase shifts in the phase stepping process, Δ*_m_*(**r**, *n*), may vary gently in space due to mild variations of the grating period from imperfect fabrication, or slight bending and misalignment of the gratings. The requirement means that the spatial scale of such variations is larger than the fringe periods.

Additionally, the fringes may be severely degraded in highly absorbing or scattering parts of the object, which renders the Fourier method ineffective in these areas. The solution we propose is to acquire a reference data set without any sample in order to obtain a template of the applied phase shifts in the phase stepping process. Then in imaging the samples, the measured phase shifts are compared to the templates, and a correction is added to account for drifts in the system that may occur between the sample and reference acquisition. The correction is in the form of a linear function of spatial coordinates. It is determined by a least-squares fitting of the difference between the measured and template phase shifts in areas where the fringe visibility is above a threshold.

The implementation follows the derivation of the Fourier analysis of interferograms [5], [8], [15], [16]. In the presence of a fundamental carrier frequency **g**, a linear phase term can be separated from the sample-induced phase shift and the applied phase shift in the phase stepping process for each harmonic order *m*:
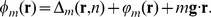
(13)


Then Eq. (4) becomes
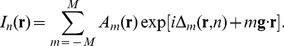
(14)


Using the definition
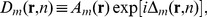
(15)it is further reduced to



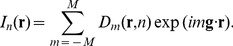
(16)The Fourier transform of the *I*_n_(**r**) is *i_n_*(**k**), given by
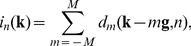
(17)with *d_m_*(**k**, *n*) being the Fourier transform of *D_m_*(**r**, *n*). By its definition in Eq. (15), *D_m_*(**r**, *n*) is typically dominated by low-spatial-frequency components, and thus, its Fourier transform, *d_m_*(**k**, *n*), is strongly peaked at zero frequency. Equation (17) means that the Fourier transform of the *n*th image, *i_n_*(**k**), is the sum of the individual Fourier transforms *d_m_*(**k**, *n*), but with each *d_m_*(**k**, *n*) shifted by a multiple of the carrier frequency *m* **g**. Thus, *i_n_*(**k**) contains multiple peaks spaced by the carrier frequency **g.** We make use of the area which is centered at a peak *m* **g** and extends half way to the neighboring peaks. This area is dominated by the Fourier transform *d_m_*(**k**, *n*). We translate this windowed area by -*m* **g** back to the center, and then inverse Fourier transform to obtain a version of *D_m_*(**r**, *n*), but with reduced resolution due to the cropped window in the Fourier domain:
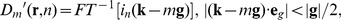
(18)with ‘ indicating that the resolution is reduced to the period of the fringes in the direction of the vector g, which is noted as eg. Since the inverse Fourier transform is preceded by translating the mth order peak back to the center, this step removes the linear phase ramp of mg·r in Eq. (13) in real space. Correspondingly, the phase of the harmonic image Dm’(r, n), noted as φm’(r, n), is a low-resolution version of the remaining contributions from the sample and the applied phase shift in phase stepping, i.e.



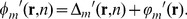
(19)As discussed earlier, the applied phase shift Δ*_m_*(**r**, *n*) only varies mildly over the length scale of the fringe period. Thus it is adequately captured by the low-resolution version Δ*_m_*’(**r**, *n*) in Eq. (19). Also by definition, the applied phase shift is relative to a particular image in the phase stepping set, e.g. the first image. Thus we can set
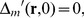
(20)and derive the phase shifts for the rest of the images from Eq. (19) as



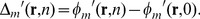
(21)At this point we reached the goal of measuring the applied phase shifts without *a priori* knowledge. The measured Δ*_m_*’(**r**, *n*) are then used in Eq. (9) and Eq. (11) to provide the linear coefficients *B_mn_*(**r**) at a reduced resolution. These are interpolated to the full detector resolution, and input into Eq. (10) to retrieve the amplitude *H_m_*(**r**) and the phase factor *φ_m_*(**r**) on a pixel-by-pixel basis, after removing the linear phase ramp *m* **g·r** arising from the carrier frequency fringes.

In imaging experiments the fringes can diminish due to attenuation or scattering in the object. In such areas the above procedure would result in noisy measurements of the applied phase shifts. The solution is to acquire a set of reference images without samples, from which the applied phase shifts Δ_r_,*_m_*’(**r**, *n*) are obtained as templates. When imaging a sample, the actual applied phase shifts may differ from the templates due to instrumental drifts. This is accounted for by adding a correction term to the template in the form of a linear function of position
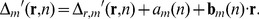
(22)


The correction term is determined from areas in the sample images where the fringes are well defined. The implementation is
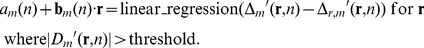
(23)


Lastly, the final results of the amplitude *H_m_*(**r**) and phase *φ_m_*(**r**) generally contains baseline contributions from instrumental factors including grating imperfections and misalignments, in addition to the linear phase ramp *m* **g·r** from the carrier frequency. These are all removed by processing the reference data set to obtain the baseline *H_r_,_m_*(**r**) and phase *φ_r_,_m_*(**r**), then removing them in the sample data according to
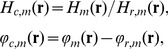
(24)with the subscript *c* indicating corrected results.

Overall, the first step of the processing algorithm is to measure the applied phase shifts in phase stepping, which comprises the calculations described by Eqs. (18–23); the second step is to retrieve the amplitude and phase of the fringes at full detector resolution, which comprises the calculations described by Eqs. (8–12), followed by the reference baseline correction of Eq. (24).

In the phase contrast imaging experiments below, the retrieved information is displayed in three images of different contrasts: the differential phase contrast image is simply the reference-corrected phase image of the first harmonic order *φ_c,1_*(**r**); the conventional intensity attenuation image is the reference-corrected amplitude of the zeroth harmonic in log scale, –ln[*H_0_*(**r**)/*H_r_,_0_*(**r**)]; the scatter (dark-field) image is the attenuation of the fringe amplitude due to scattering in excess of intensity attenuation, again in log scale as –{ln[*H_1_*(**r**)/*H_r_,_1_*(**r**)]–ln[*H_0_*(**r**)/*H_r_,_0_*(**r**)]}. For the application of scatter correction, the reference-corrected amplitude of the first harmonic in log scale, –ln[*H_1_*(**r**)/*H_r_,_1_*(**r**)], is the desired image which is free of scattered x-rays.

### Application to Phase-Contrast Imaging Using Electromagnetic Phase Stepping

#### Ethics statement

The ex vivo mouse imaging study was performed under a National Heart, Lung and Blood Institute Animal Care and Use Committee approved protocol.

Electromagnetic phase stepping is a method for phase stepping without mechanical motion. In the presence of a carrier frequency, the essential requirement for phase stepping is a relative movement between the fringes and the projection image of the object. EPS achieves the condition by electromagnetically shifting the focal spot of the x-ray tube in a transverse direction across the fringe pattern, e.g. with an externally applied magnetic field that deflects the electron beam in the x-ray tube (Fig. 1B). Shifting the focal spot causes an opposite movement of the projection of the object on the detector plane, while the fringes can be made to remain stationary or move by a different amount. For example, in the case where a single grid is placed in front of the object for scatter (dark-field) imaging or scatter correction [5], [6], the fringes are displaced by larger shifts when compared with the projection image. In the case of the three-grating Talbot-Lau interferometer for phase contrast imaging [17], the movement of the fringes is controlled by the arrangement of the gratings, and can be either null (as illustrated by imaging experiments in this study), or larger than the projection of the object. In all cases, the images are digitally shifted back to align the projections of the object. The result is that the fringes move over a stationary projection of the object, effectively synthesizing the phase stepping process.

In EPS the shift of the focal spot scales proportionally with the applied magnetic field according to the action of the Lorenz force on the electron beam in the X-ray tube. The magnetic field is generated by the applied electrical current into a solenoid coil (Fig. 1B), which is set at pre-programmed levels. The amount of the focal spot shift as a linear function of the applied current is determined in a calibration procedure by measuring the opposite movements of the projections of small tungsten beads on the image plane under different current levels. Through the calibration procedure the shift for a given applied current is known to an accuracy of 0.01 mm (10 µm) or better. The response time of the focal spot movement is the time it takes to switch the magnetic field in the solenoid coil, which is the time constant of the coil. It is set by the inductance and resistance of the coil, and was 200 µs in our setup. Thus, the response time of the focal spot movement was 200 µs in our experiments. The amount of movement of the projection image depends on where the object is situated along the optical axis from the source to the detector. As a result, the digital alignment process is specific for a plane (the focal plane) along the optical axis. For a thick object, a single data set can be used in separate processing runs for a series of focal planes which focuses on different sections of the object.

We applied the APD phase stepping algorithm to phase-contrast imaging using EPS in a three-grating Talbot-Lau interferometer [17]. The imaging device (Fig. 1B) employed of a tungsten-target x-ray tube operating at 55 kVp/1 mA with a focal spot size of approximately 50 µm, and an x-ray detector with a pixel size of 30 µm and a matrix size of 2048×2048. The interferometer consisted of three gratings of 4.8 µm period with the first and third being intensity gratings (Microworks GmbH) and the second being a phase grating. All gratings were rotated around the vertical axis by 45° to increase the effective depths. The gratings were positioned at equal spacing over a total distance of 76 cm. The third grating was slight rotated around the optical axis to create vertical moiré intensity fringes of 290 µm period. In this particularly way of creating the moiré fringes, the fringe pattern is independent of the position of the focal spot of the cone beam, and remains stationary during electromagnetic phase stepping. For EPS a home-made copper solenoid coil was attached to the front surface of the x-ray tube housing to generate a magnetic field in the tube. The coil was driven by a digital power supply which provided up to 2.0 A of current at up to 8 W of power. The corresponding peak magnetic field was 3.1 mT at the location of the electron beam inside the x-ray tube. The field from a 1.5 A current was sufficient to shift the focal spot by 380 µm in the horizontal direction, perpendicular to the moiré fringes. The deflections of the focal spot at various levels of input current into the coil were known from calibration measurements. Each EPS set comprised 6 images of increasing current levels from 0 to 1.5 A.

## Results

**Figure 2:**
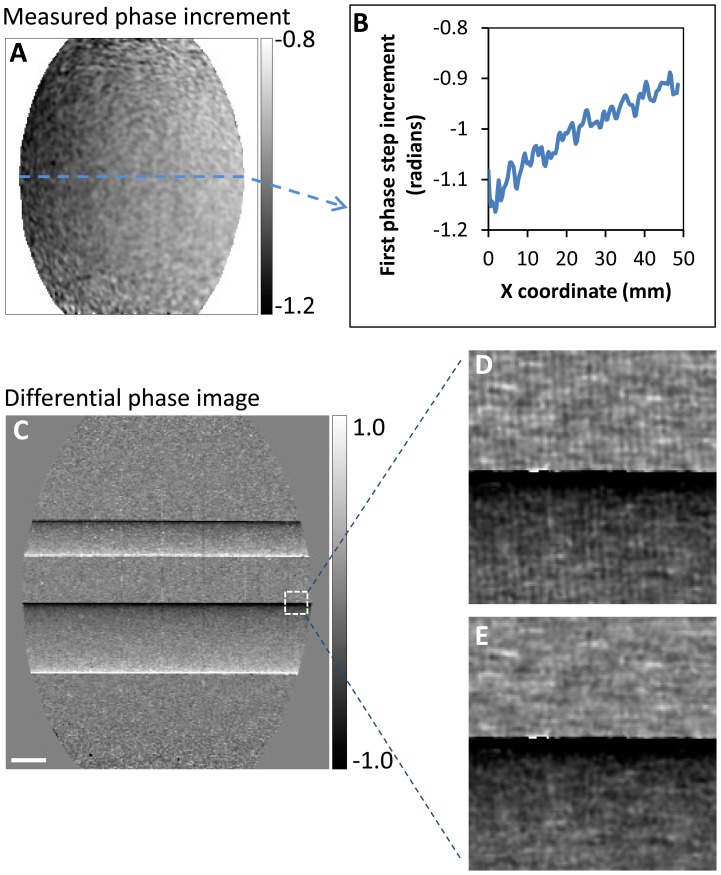
The arbitrary and position dependent (APD) phase stepping algorithm improves phase retrieval. (**A**) In an example of phase contrast imaging with electromagnetic phase stepping, the measured phase increment in the first of 6 steps is shown. Considerable variation can be seen over the oval area covered by the gratings. A profile across the center of the area (**B**) revealed a 20% gradual decrease of the phase increment. (**C**) For comparison, the differential phase contrast image of two horizontal polyacetal rods was retrieved with both the APD and the previous globally uniform algorithms. In the area outlined by the small square, the previous algorithm resulted in vertical fringe artifacts (**D**) which indicate incomplete demodulation of the moiré fringes, while the APD algorithm removed the artifacts (**E**). The scalebar in (**C**) is 3 mm long.

We compared the APD algorithm with the previous algorithm assuming globally uniform phase steps by Goldberg and Bokor [15]. A sample consisting of two horizontal polyacetal plastic rods was imaged for the comparison. The phase increments measured by the APD algorithm showed variations with position, in a peak-to-peak range of 20% of the global mean over the area covered by the gratings (Fig. 2A, B). The global mean phase increments among the 6 phase steps varied from −0.979 to 1.020. When spatially uniform phase increments were assumed, the retrieved differential phase maps contained residual fringe artifacts in the areas where the phase increment deviated from the global mean value (Fig. 2D). The artifacts represent incomplete demodulation of the carrier frequency fringes. When the APD algorithm was used, the artifacts were eliminated and the fringe demodulation was complete in the entire grating area (Fig. 2E).

**Figure 3:**
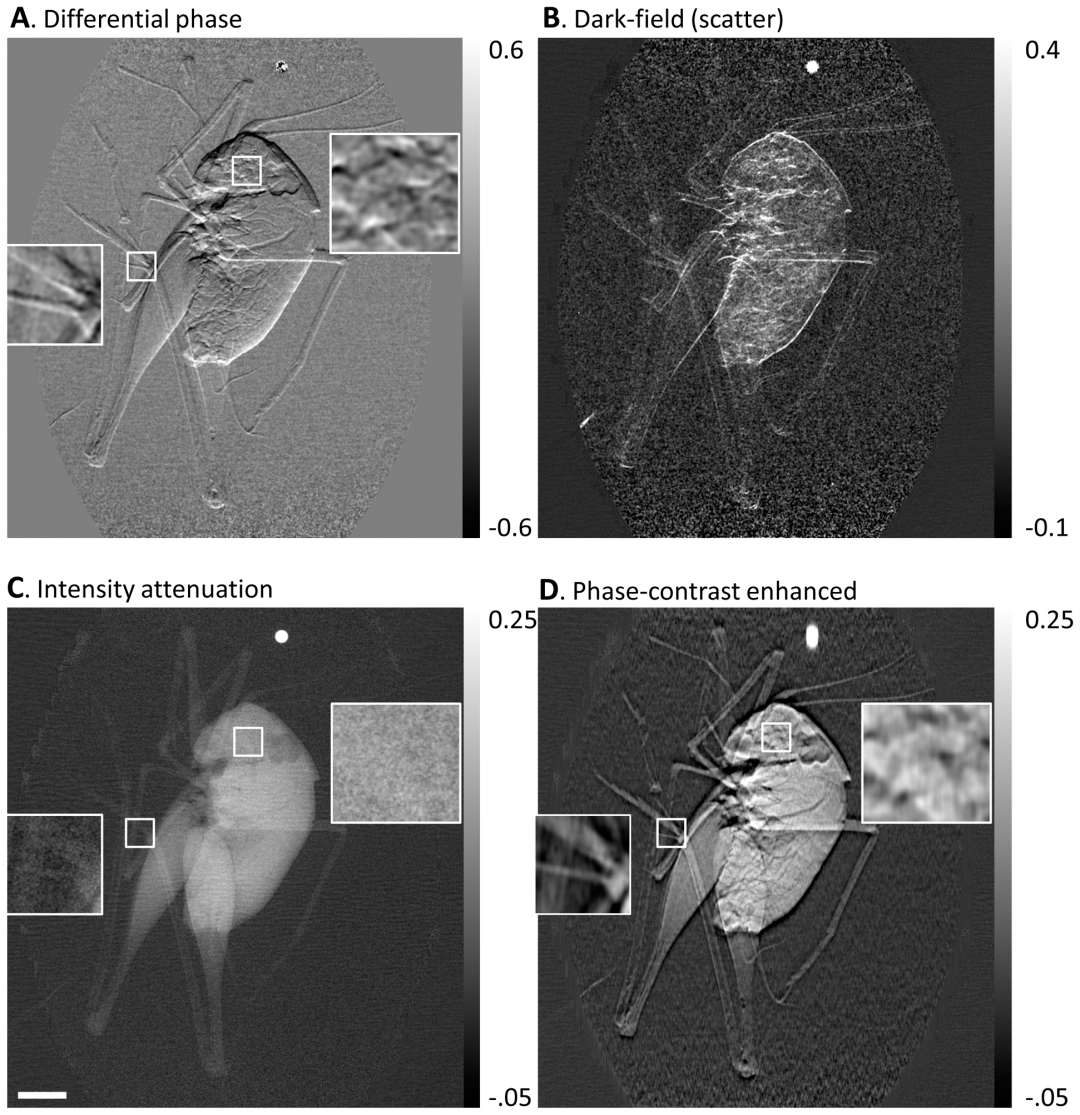
Retrieved images of a cricket from an electromagnetic phase stepping set. The arbitrary and position dependent phase stepping algorithm was used to calculation (**A**) the differential phase, (**B**) the scatter (dark-field), (**C**) the conventional attenuation, and (**D**) the phase-contrast enhanced images. The phase-contrast enhanced image combines the high spatial frequency information of the differential phase image with the low spatial frequency information of the attenuation image. The bright dot above the cricket is a tungsten bead. Two small areas in the leg and head of the cricket are outlined with white squares and shown in magnified view. The details seen in the phase contrast images (**A**) and (**D**) are absent in the conventional attenuation image (**C**). The scalebar in (**C**) is 3 mm long.

An example of applying the APD algorithm to biological samples was demonstrated in an imaging experiment of a cricket. A single set of data from the electromagnetic phase stepping procedure was used to retrieve several types of contrasts, including the differential phase contrast, the scattering or dark-field, and the conventional attenuation contrast (Fig. 3). A phase-contrast enhanced (PCE) image was also obtained by combining the low spatial frequency information of the attenuation image and the high spatial frequency information from the differential phase contrast [18], [19] (Fig. 3). The PCE image shares the same global features with the conventional attenuation image but with more visible details at smaller length scales.

**Figure 4:**
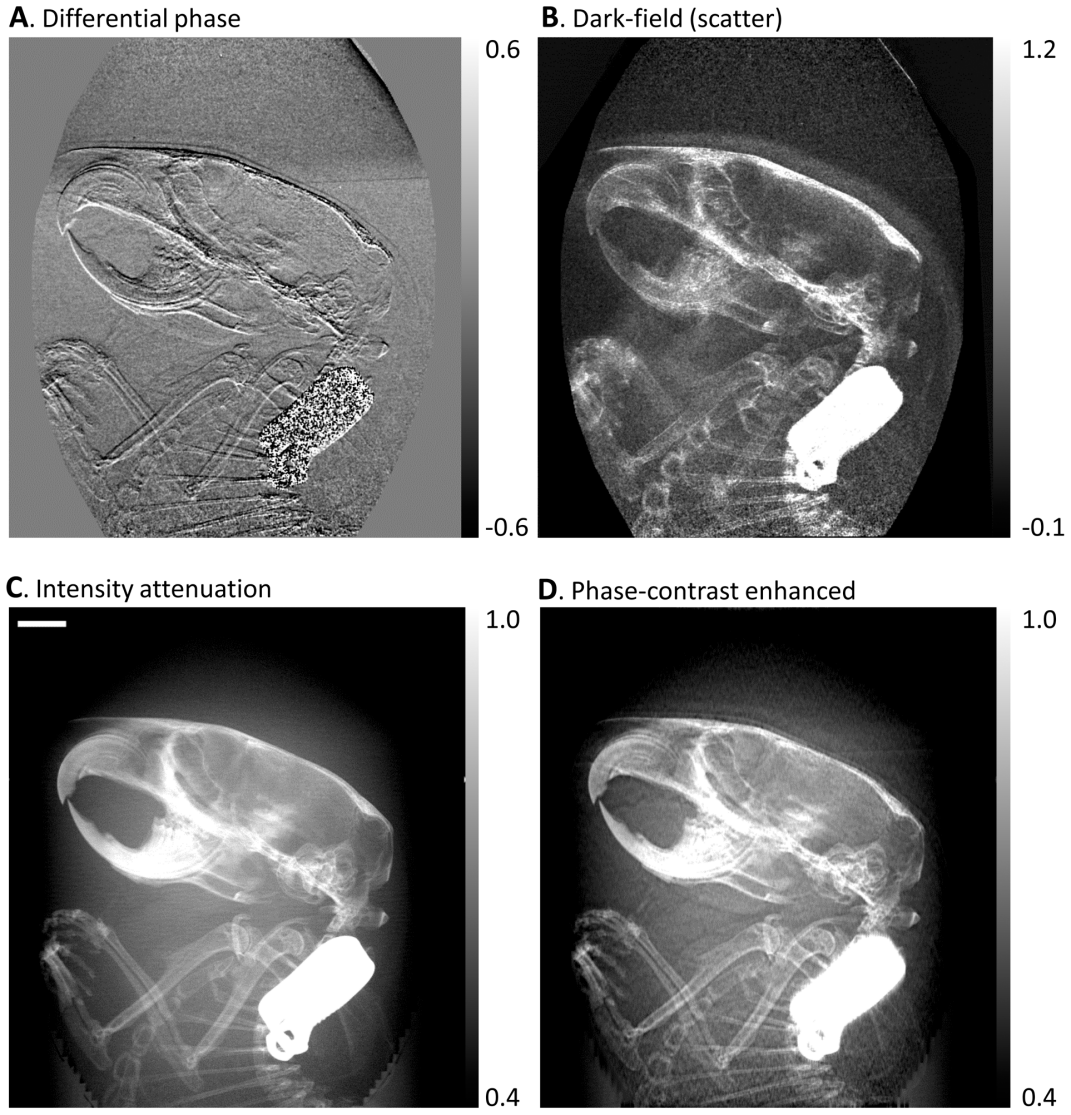
Retrieved images of the head region of a mouse. The body of the mouse was fixed in formalin and then immersed in water in a plastic tube. Sagittal projections of the head and thorax area were taken. The arbitrary and position dependent phase stepping algorithm was used to analyze an electromagnetically phase stepped set of images. The results are (**A**) the differential phase contrast, (**B**) the dark-field (scatter), (**C**) the intensity attenuation and (**D**) the phase-contrast enhanced images (defined in Fig. 3). The beak-like structures in the top left are the upper and lower jaws and teeth of the mouse. The front legs can be seen below the skull. The bright rectangular object is a metallic ID tag. Phase contrast brings forth soft tissue details that are missing in the attenuation image. The scalebar in (**C**) is 3 mm long.

A further example of a biological application was an imaging study of a formalin fixed body of a mouse under an institutional IACUC approved protocol (C57BL/6 wild-type, 5 year old male). A sagittal projection of the head and chest region of the mouse was acquired. The three types of contrasts along with the phase-contrast enhanced image are shown in Fig. 4. The value of phase contrast lies in the enhanced high-spatial-frequency details that are visible in the differential phase contrast and the phase-contrast enhanced images but are either absent or less visible in the conventional attenuation image.

## Discussion

By introducing a high-spatial-frequency modulation into the propagating wave of an x-ray imaging system using grids or gratings, both the scattering and refraction of the wave can be quantified at the full resolution of the detector through the phase stepping procedure. This then allows for phase-contrast [2], [4] and scatter imaging [5], [7] as well as removal of the “fog” of diffusely scattered x-rays for improved image clarity [3], [6]. In less than ideal experimental conditions as well as practical application settings, both mechanical and electromagnetic phase stepping procedures can bring about phase increments that vary from step to step and also spatially from location to location in the field of view [20]. We showed that the APD algorithm can effectively deal with such conditions, and is particularly well suited for the implementation of electromagnetic phase stepping. Although the experimental tests were performed with x-ray, the algorithm traces its lineage back to optical wavefront measurements and can be directly applied there.

The APD algorithm involves more computation than previous algorithms that assume ideal or uniform phase increments. We found that the computation time for each data set was approximately 30 seconds on a 2008 model laptop PC using a home-made software. The software was written in the IDL data processing language (Exelis Visual Information Solutions, Inc). Thus, it should be possible to perform image processing in near real time with modern workstations.
